# Draft genome sequencing of multidrug-resistant *Escherichia coli* strain AHSTU04 isolated from bovine milk in China

**DOI:** 10.1128/mra.00570-26

**Published:** 2026-07-01

**Authors:** Muhammad Haris, Wang Zekai, Hasnain Ali Khan, He Shaojun

**Affiliations:** 1 College of Animal Science, Anhui Science and Technology University, Fengyang, Anhui, China; Fluxus Inc., Sunnyvale, California, USA

**Keywords:** *Escherichia coli*, bovine mastitis, whole-genome sequencing

## Abstract

We report the draft genome sequence of multidrug-resistant *Escherichia coli* strain AHSTU04, isolated from bovine raw milk in Anhui, China. The 4.96-Mbp genome shows phenotypic resistance to oxacillin and other antibiotics via disk diffusion, harbors 189 virulence factors, lacks plasmids, and acquired antimicrobial resistance genes, suggesting that resistance is intrinsic.

## ANNOUNCEMENT

*Escherichia coli* causes clinical and subclinical mastitis in dairy cows, leading to financial losses for the dairy industry owing to lower milk production, early slaughter, and medical expenses (1, 2). Through repeated exposure to antibiotics, it frequently acquires antimicrobial resistance and can serve as a potential vehicle for zoonotic transmission of antimicrobial resistance through the food chain (3, 4). In this study, we isolated and characterized a multidrug-resistant *E. coli* strain AHSTU04 from raw milk samples collected from lactating cows at a commercial farm in Bengbu city, Anhui, P.R. China (32.92° N, 117.39° E).

To isolate *E. coli* strain AHSTU04, two milk samples (10 mL) that had been aseptically collected on 5 February 2026 in sterile tubes were transported inside an icebox (<4°C) to the laboratory. A 200 µL aliquot of the milk sample was placed in 5 mL of nutrient broth (Hopebio, China) and aerobically incubated overnight at 37°C. The enriched broth was then streaked onto MacConkey agar, and after 24 h of incubation at 37°C, presumptive colonies based on Gram staining (gram-negative), colony morphology, and biochemical tests (citrate, xylose, fructose, inositol, maltose, galactose, rhamnose, glucose, sucrose, arabinose, sorbitol fermentation, and urease activity) were subcultured to get pure isolates. Antibiotic resistance was determined through the disk diffusion method on Mueller-Hinton agar (HopeBio, China) against a panel of 11 antimicrobial agents (Hangwei Hangzhou, China) following the Clinical and Laboratory Standards Institute guidelines (5).

Genomic DNA was extracted from a fresh culture grown on nutrient agar for 16–18 h at 37°C using the MagPure Bacterial DNA Kit (D6361-02, Magen, China) (6). DNA concentration was determined with a Qubit 4.0 fluorometer (Q33226, Thermo Fisher, USA), and integrity was assessed by 1% agarose gel electrophoresis. A paired-end library with an insert size of 200–400 bp was prepared using the Hieff NGS Ultima Pro DNA Library Prep Kit (Yeasen Biotechnology, Shanghai, China) without modifications and sequenced on the MGI DNBSEQ-T7 platform (Sangon Biotech, Shanghai, China). Fastp (version 0.23.0) was used to quality-filter raw reads, and *de novo* assembly was performed via SPAdes (v 3.5.0)(7, 8). GapFiller (v 1.11) and Pilon (v 1.23) were then used to fill in gaps and correct errors (9, 10). BUSCO was used to assess genome completeness, and the National Center for Biotechnology Information Prokaryotic Genome Annotation Pipeline (v 1.2.1) was used for annotation (11). CARD (v 3.2.0) and ResFinder (v 4.1) were used to identify antimicrobial resistance genes (12, 13); VFDB was used to identify virulence factors. PlasmidFinder (v 2.1) was used to screen plasmid replicons (14). Using Genome-wide Genome BLAST Distance Phylogeny (GBDP) and Average Nucleotide Identity (ANI) analysis on JSpeciesWS, the Type Genome Server (TYGS) verified species identification (15, 16); ClermonTyping determined the phylogroup (17). Default parameters were used for all software unless otherwise specified.

The draft genome of *E. coli* strain AHSTU04 (phylogroup B1) showed 100% completeness and harbored 189 virulence genes, with no plasmids or acquired antimicrobial resistance genes detected, despite phenotypic resistance to multiple antibiotics (Table 1). TYGS analysis confirmed species identification (dDDH 74.6% against *E. coli* DSM 30,083).

**TABLE 1:** Genomic features of *E. coli* strain AHSTU04 isolated from bovine raw milk^*a*^

Full name	*Escherichia coli* AHSTU04
Species identification	*Escherichia coli*
Accession no.	JBXVQE000000000
SRA no.	SRR38241559
Submission no.	SAMN57437756
Project no.	PRJNA1456959
Closest neighbor (ANIb% by JSpeciesWS)	*Escherichia coli* CHS 77 (GCA_000692735.1; 99.468%)
Total read count	7,557,954
Total bases (bp)	1,133,693,100
Read length (bp)	2 × 150
Total genome size (bp)	4,962,390
No. of contigs	54
Genome completeness (%)	100
GC (%)	53.49
Coverage (×)	228
Q20 (%)	97.93
Q30 (%)	96.44
L50	5
N50	383,113
N90	89,942
No. of total genes	4,923
No. of coding sequences (CDS)	4,816
No. of tRNA	84
No. of rRNA	9
No. of ncRNAs	11
No. of pseudo genes (total)	120
CRISPR arrays	2
No. of virulence genes	189
PathogenFinder score	0.9801
No. of plasmids	0
Phenotypic resistance profile (zone diameter, mm; interpretation)	Oxacillin (0, R), kanamycin (8, R), cephalexin (17, R), erythromycin (11, R), clindamycin (0, R), ampicillin (14, I)

^*a*^ Sequencing coverage of 228× was calculated as the total number of sequenced bases (1,133,693,100 bp) divided by the assembled genome size (4,962,390 bp).

## Data Availability

The whole-genome shotgun project of the *E. coli* strain AHSTU04 has been deposited in GenBank and the NCBI Sequence Read Archiveunder BioProject accession number PRJNA1456959. The version described in this paper is version JBXVQE000000000.1.
